# D/H ratios of the inner Solar System

**DOI:** 10.1098/rsta.2015.0390

**Published:** 2017-04-17

**Authors:** L. J. Hallis

**Affiliations:** School of Geographical and Earth Sciences, Gregory Building, University of Glasgow, Glasgow G12 8QQ, UK

**Keywords:** hydrogen isotopes, water, meteorites, terrestrial planets, asteroid belt

## Abstract

The original hydrogen isotope (D/H) ratios of different planetary bodies may indicate where each body formed in the Solar System. However, geological and atmospheric processes can alter these ratios through time. Over the past few decades, D/H ratios in meteorites from Vesta and Mars, as well as from S- and C-type asteroids, have been measured. The aim of this article is to bring together all previously published data from these bodies, as well as the Earth, in order to determine the original D/H ratio for each of these inner Solar System planetary bodies. Once all secondary processes have been stripped away, the inner Solar System appears to be relatively homogeneous in terms of water D/H, with the original water D/H ratios of Vesta, Mars, the Earth, and S- and C-type asteroids all falling between δD values of −100‰ and −590‰. This homogeneity is in accord with the ‘Grand tack’ model of Solar System formation, where giant planet migration causes the S- and C-type asteroids to be mixed within 1 AU to eventually form the terrestrial planets.

This article is part of the themed issue ‘The origin, history and role of water in the evolution of the inner Solar System’.

## 1. D/H ratio models of the early Solar System

Theoretical studies of interstellar chemistry show that in astrophysical environments, at temperatures less than 50 K, water becomes enriched in deuterium (^2^H or D) relative to molecular hydrogen, with D/H ratios reaching 0.001–0.01 [1–5]. These theoretical studies are supported by astronomical observations of D-rich water (D/H = 0.001–0.08) in the envelopes surrounding protostars [6,7]. Thus, prior to the formation of our Sun, water in the molecular cloud would have had a high D/H ratio. However, when this D-rich water is incorporated into the hot inner region of a protoplanetary disc, isotopic exchange reactions occur with other hydrogen-bearing species (e.g. H_2_O + HD ↔ HDO + H_2_), which dramatically lower the D/H ratio (e.g. [8–15]). Isotopic exchange occurs more rapidly at high temperatures, meaning water in the inner regions of the disc would equilibrate with H_2_ gas, producing low D/H ratios of approximately 2 × 10^–5^ [16]. By contrast, because isotopic exchange is sluggish at low temperatures, water in the outer regions of the disc would preserve its original high D/H ratio from the molecular cloud. Therefore, after the Sun's formation the D/H ratio of water in the evolving protoplanetary disc would have been dependent (at least initially) on the temperature of the surrounding environment, and thus distance from the Sun.

Measurements of D/H ratios in Oort cloud comet (OCC) water are highly variable (1.4–6.5 × 10^−4^), with the lowest measured ratio being similar to terrestrial ocean water [17–29]. The Jupiter family comets (JFCs) Hartley 2 and 45P/Honda–Mrkos–Pajdušáková have low water D/H ratios (1.61 and less than 2.0 × 10^−4^) [30,31], whereas the ROSINA mass spectrometer aboard the Rosetta spacecraft measured a high D/H ratio (three times that of the Earth's oceans) in water vapour sublimated from the JFC 67P/Churyumov–Gerasimenko [32]. The overlap in D/H ratios, along with other indistinguishable chemical and physical characteristics [33], argues for a common parent population for the OCCs and JFCs [34]. However, D/H ratio variation implies the parent population formed over a long period of time or distance from the Sun. As an added complication, all these measurements are of sublimated water from the surface of comets, thus probably do not represent the bulk comet D/H ratio.

**Figure 1.**
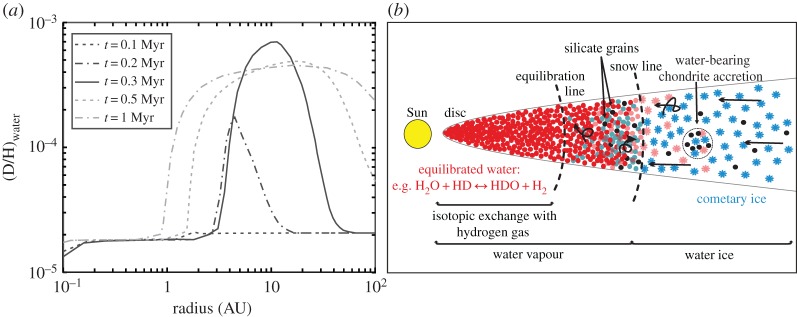
D/H ratio evolution in the early Solar System. Isotopic equilibration with molecular hydrogen results in a low water D/H ratio in the hot inner disc (less than 2 AU) for the first approximately 0.3 Myr after the Sun's formation (*a*; adapted from [16]). By contrast, outer disc water (approx. 2–40 AU) remains unequilibrated, retaining the high D/H ratios inherited from the molecular cloud. Beyond approximately 0.3 Myr turbulent mixing produces a more uniform D/H distribution in the outer disc, with some molecular-hydrogen-equilibrated water being transported beyond the snowline to the region where the chondritic meteorites formed (*b*; adapted from [35]).

Yang *et al*. [16] coupled a dynamic model of material transport and mixing with a kinetic study of D–H isotopic exchange in an attempt to explain the variation in cometary D/H ratios, and to produce a picture of the first 1 Myr of Solar System history (figure 1*a*). These authors found that, during the very earliest stages of the protoplanetary disc (approx. 0.1 Myr after formation), viscous spreading may have redistributed low D/H ratio water from the inner to the very outer disc regions (figure 1*a*). Inside of 2 AU the D/H ratio of water is shown to be almost equal to that of molecular hydrogen. Viscous spreading would have redistributed this low D/H water throughout the protoplanetary disc by 0.1 Myr. At a later stage (approx. 0.2 Myr), molecular cloud infall begins to add high D/H water to the disc. In the inner disc, this molecular cloud water exchanges with molecular hydrogen, and the D/H ratio remains low. Beyond 2 AU low temperatures prevent this exchange and the D/H of water reflects the high ratio of the molecular cloud, with maximum values at approximately 3 AU and 10 AU in the 0.2 Myr and 0.3 Myr snapshots, respectively (figure 1*a*). However, this infall does not affect the outermost part of the disc, hence the D/H ratio of water remains low in this region. Therefore, observed cometary D/H variation can be explained if the parent population contained material from both the outer low D/H region and the approximately 2–10 AU high D/H region.

Beyond 0.3 Myr after the formation of the protoplanetary disc, once molecular cloud infall stops, turbulent mixing results in a more uniform D/H ratio in the outer disc. Gas turbulence would also result in some of the low D/H water from the inner disc being transported beyond the snowline, to the region where the chondritic meteorites are thought to have formed (figure 1*b*). Therefore, water in these meteorites should be composed of both an H_2_ equilibrated water component from the inner disc and a cometary water component that drifted inwards from the outer disc [35]. Transport and mixing would have been particularly efficient in the early stages of disc evolution, if the disc was first built compact and then expanded because of turbulence [36]. This expansion is consistent with the presence of crystalline silicates in comets [20].

## 2. D/H ratio measurements

D/H ratios are commonly quoted relative to Vienna Standard Mean Ocean Water (VSMOW; D/H = 1.5576 × 10^−4^) using the notation δD = {[(D/H)_unknown_/(D/H)_VSMOW_] − 1} × 1000, in units of parts per thousand (per mil (‰)). This means VSMOW has a δD value of 0‰. Deuterium-enriched reservoirs have high δD values (e.g. interstellar water δD ≈ +10 000‰ [37]), and D-poor reservoirs have low values (e.g. protosolar disc δD ≈ −870‰ [38]). This notation will be used in the following sections to compare the D/H ratios of various different extraterrestrial materials.

### (a) Eucrites

Eucrites belong to the howardite–eucrite–diogenite group of meteorites that are derived from the asteroid belt, dominantly from the asteroid Vesta [39–41]. These basaltic meteorites are some of the oldest igneous rocks in the Solar System, mostly having crystallized only approximately 8–20 Myr after the first solids formed [42,43]. Sarafian *et al*. [44] measured the D/H ratio of structurally bound water in eucrite apatite [Ca_5_(PO_4_)_3_(OH,F,Cl)], reporting δD values of between −231‰ and −37‰ from five different meteorites. The authors state that these values probably reflect the original D/H ratio of the parent body (Vesta). As the apatite grains have young cosmic ray exposure ages, high water contents and a small range of D/H ratios across the different exposure ages and metamorphic grades, contamination by exogenic H from solar wind and/or by D produced during spallation processes is unlikely [44]. Degassing could have raised the D/H ratio from its original value, because of the preferential evaporation of the lighter hydrogen isotope. However, the small observed spread in δD with the relatively large spread of water contents in the analysed apatite indicates that degassing did not occur in these meteorites [45,46]. Stable isotope fractionation between apatite and melt is expected to be small (approx. 20‰) at high temperatures; thus, apatite-melt fractionation should be minor relative to the variation in δD observed [47]. Therefore, none of these three processes substantially affected the H isotopic compositions measured [44]. As each apatite grain is stoichiometric, hydrogen contamination via terrestrial weathering is ruled out.

### (b) Carbonaceous chondrites

Reported bulk rock D/H ratios for the carbonaceous chondrites vary between δD values of +2150‰ and −229‰ [48,49]. However, these values are not representative of chondritic water alone, as the bulk rock includes hydrogen within insoluble organic material (IOM). Hydrous phyllosilicate is present in the CI, CM and Tagish Lake carbonaceous chondrites, as well as in a number of CR and CV meteorites. However, it is intergrown with IOM at the micrometre scale, meaning physical separation is not possible. Alexander *et al*. [49] calculated the D/H ratio of water in the CM and CR chondrite groups by comparing the bulk rock D/H and C/H ratios of group members showing different degrees of aqueous alteration. Assuming the bulk hydrogen isotopic compositions are produced by simple mixing of hydrated silicates and IOM, the bulk compositions should form a line on a plot of δD versus C/H, with the hydrogen isotope intercept giving the isotopic composition of water. The CM and CR chondrites do form a line on this type of plot, producing calculated water D/H ratios of −444 ± 23‰ and +96 (+110/−65)‰, respectively [49].

The CI chondrites, along with the most primitive CO and CV chondrites, are not common enough to use the above approach. However, the CI's Orgueil and Ivuna, along with the CO ALH 77307, fall on the CM trend, suggesting that they all probably had similar initial water compositions (table 1). If all chondrites accreted a common IOM component [60], chondrite water contents can also be calculated via subtraction of this component. This method is especially useful for those C-chondrite groups with only one or a few members (CIs, COs, CVs and Tagish Lake). By applying this methodology, Alexander *et al*. [49] calculated that C-chondrite water has current D/H ratios between δD −587‰ and +207‰ (table 1).

**Table 1.** The D/H ratio of inner Solar System water reservoirs.

	D/H (×10^−4^)	δD (‰)	reference
protosolar disc	0.21 ± 0.04	−863 to −868	[38,50]
the Earth			
bulk Earth	1.49 ± 0.03	−24 to −62	[51]
VSMOW	1.56	0	[52]
GISP	1.26	−190	[53]
MORB	1.46–1.47	−55 to −65	[54]
deep mantle	<1.22	<−218	[55]
Mars			
interior	<1.99	<275	[56–58]
atmosphere	7.58–10.90	4950 ± 1080	[59]
Vesta	1.2–1.5	−231 to −37	[44]
C-chondrite water			
CI	0.64–0.98	−373 to −587	[49]
CM	0.83–0.90	−421 to −468	[49]
CO	0.85–1.32	−152 to −455	[49]
CR	1.61–1.88	34–208	[49]
CV	≤0.82	≤−473	[49]
Tagish Lake	≤1.14	≤−268	[49]
O-chondrite water			
Semarkona	2.80–3.44	798–1209	[49]
R-chondrite water	7.26 ± 0.13	3579–3746	[49]

### (c) Non-carbonaceous chondrites

High D/H ratios are present in ordinary chondrite (OC) IOM, and these ratios increase with increasing metamorphism, to values as high as δD 12 000‰ [60,61]. In addition, the D/H ratio of water in the OC Semarkona is much higher than the ratios of the carbonaceous chondrites (δD = 798–1209‰ [49]). These high D/H ratios appear to be a product of oxidation in the meteorite, rather than a reflection of the original parent body D/H ratio—water becomes isotopically heavy due to the oxidation of Fe and the loss of isotopically very light H_2_. This heavy water then exchanges with the IOM.

The Rumuruti (R) chondrites are highly oxidized, containing rare or no Fe–Ni metal, as well as abundant ferromagnesian minerals rich in Fe^3+^ (e.g. [62,63]). Direct measurements of water D/H ratios are possible in the R-chondrite LaPaz Icefield (LAP) 04840, because it (uniquely) contains abundant OH-bearing silicate minerals (approx. 13% ferri-magnesiohornblende and approx. 0.4% phlogopite by volume), as well as rare apatite [63]. The D/H ratio of water in LAP 04840 hornblende and phlogopite ranges from δD 3595‰ to 3743‰ and from δD 2739‰ to 3043‰, respectively (table 1). As with the OCs, oxidation of Fe by water could also produce such D enrichments in the remaining water. Oxidation also destroys IOM, releasing D-rich hydrogen into the chondrite matrix. This hydrogen is subsequently incorporated into hornblende and phlogopite during their metamorphic formation [63]. R-chondrites are more oxidized (near the quartz–fayalite–magnetite buffer) than the OCs, which explains their higher water D/H ratios [63].

### (d) Mars

In contrast to the asteroidal parent bodies of the eucrites and chondrites, Mars is large enough to have retained a substantial atmosphere. Therefore, it has at least two separate hydrogen isotope reservoirs (possibly three; see [64]). The atmospheric reservoir and the interior mantle reservoir are completely separated, because Mars has no plate tectonics. Therefore, there has been no recycling of atmospherically equilibrated crustal material back into the mantle.

The current Martian atmosphere is deuterium enriched, with ratios ranging from δD 0–2000‰ in high-altitude regions to δD 7000‰ in the polar regions [65]. The D/H ratio of Mars' atmosphere at Gale Crater, measured *in situ* by the Mars Curiosity rover, was reported as δD 4950 ± 1080‰ [59]. These high D/H ratios are caused by the preferential loss of the lighter hydrogen isotope to space via Jeans escape [66]. Hence, over geological time the D/H ratio of the Martian atmosphere is expected to have increased.

High atmospheric D/H ratios are represented in Martian meteorites via secondary alteration minerals. The nakhlite group of meteorites contain Martian aqueous alteration assemblages, consisting of smectite phyllosilicate, siderite carbonate, sulfates, Fe-oxides and hydroxides, and halite (e.g. [67–79]). These assemblages have been reported to contain high D/H ratios (up to δD 1165‰ [56]), although wide variability highlights the ease of isotopic exchange between certain alteration minerals and the terrestrial atmosphere. However, the maximum value in this case provides a minimum for the Martian atmosphere at the time of alteration formation (633 Ma [80]). Allan Hills (ALH) 84001 is the most ancient Martian meteorite, having crystallized at approximately 4.1 Ga (e.g. [81,82]). This orthopyroxenite contains small carbonate rosettes (less than 200 µm diameter), which are zoned from Ca and Fe rich to Mg rich [83–87], and are reported to have formed at approximately 3.9 Ga [88]. D/H ratios vary in these carbonates between δD 182‰ and 2092‰ [89,90]. This variation may be caused by different levels of terrestrial contamination, which would drag the D/H ratio down towards 0‰. Alternatively, or perhaps in addition, the high shock pressures experienced by this meteorite [86,91–93] could have caused shock implantation of atmospheric hydrogen into carbonate, driving the D/H ratio higher in the most shocked grains (see below).

**Figure 2.**
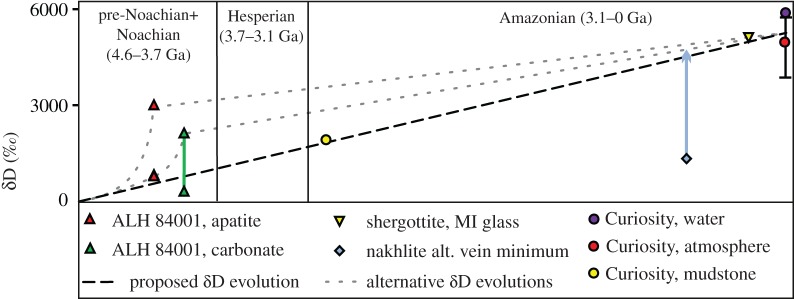
The evolution of D/H ratio in the Martian atmosphere over time. Previous estimates of atmospheric D/H increase used ALH 84001 apatite D/H to suggest a rapid initial hydrogen (and general atmospheric) loss prior to 4.1 Ga (e.g. [65]). However, as ALH 84001 is a highly shocked meteorite that is reported to contain a crustal assimilant, the D/H ratio of its apatite may not be representative of the Martian atmosphere at the time of crystallization.

**Figure 3.**
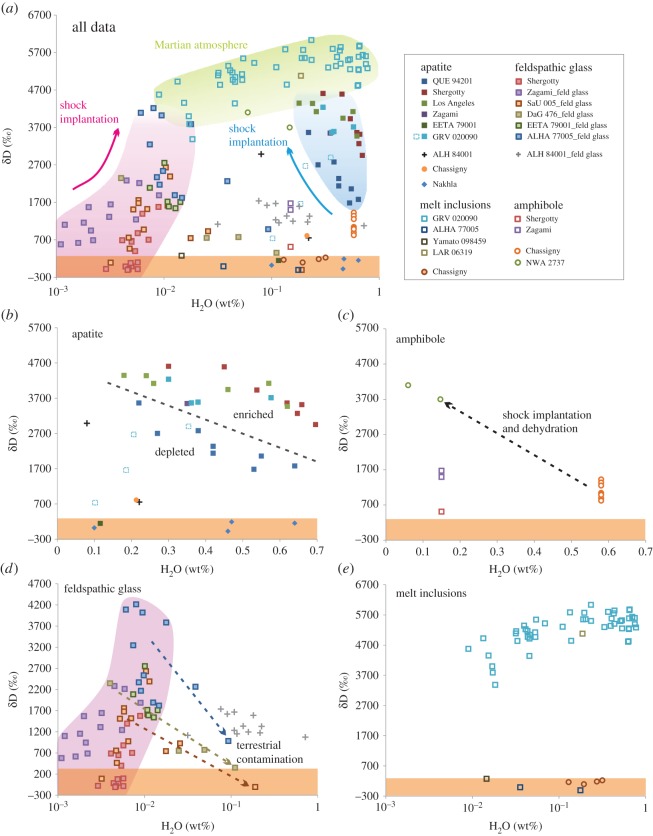
(*a*–*e*) D/H ratio (δD) versus water content in Martian igneous minerals. High-impact shock pressures, mantle source enrichment and crustal assimilation can add D-rich atmospheric hydrogen to igneous minerals, meaning the majority do not have D/H ratios representative of the Martian mantle (δD < 275‰, orange envelope). See the electronic supplementary material for data and references.

If meteorite alteration mineral D/H ratios are plotted in conjunction with current atmospheric measurements, as well as *in situ* lithological measurements from the Curiosity rover [94], a picture of atmospheric loss over time is produced (figure 2). These data suggest a linear increase in the atmospheric D/H ratio with time, and an origin of approximately 0‰. This low initial D/H ratio supports a volcanic origin for Mars' atmosphere, because measurements of melt inclusion (MI) glass and apatite in the most primitive and least shocked Martian meteorites indicate that the D/H ratio of Mars' mantle is low (δD < 275‰ [57,58]). However, not all hydrous primary (igneous) minerals contain D/H ratios representative of the Martian mantle reservoir (figure 3). In fact, the majority of these minerals have D/H ratios somewhere between low mantle and high crustal/atmospheric ratios (assuming the crust and atmosphere are equilibrated, but see [64]). There are a number of ways that these intermediate D/H ratios can be produced, both pre- and post-crystallization.

In contrast to eucrite apatite D/H ratios, apatite in the shergottites and ALH 84001 shows a distinct trend of increasing D/H with decreasing water content (figure 3*b*). This trend is also weakly apparent in ALH 84001 feldspathic glass (figure 3*d*), and is characteristic of H_2_ degassing from a melt [45,46]. By contrast, Nakhla apatite D/H ratios stay constantly low over a range of water contents, indicating a lack of degassing in this melt. These low D/H ratios probably represent the Martian mantle reservoir.

A similar D/H trend is visible in chassignite amphiboles (figure 3*c*). However, instead of each meteorite exhibiting a range of D/H and water contents, two separate chassignites display very different amphibole compositions [95]. The high shock pressures experienced by meteorites upon ejection from the Martian surface may affect their isotopic ratios, especially for volatile elements. Experimental studies have shown that shock devolatilization of amphibole decreases its water content while simultaneously increasing the D/H ratio, because the lighter isotope is preferentially lost [96,97]. Hydrogen implantation from the surrounding atmosphere was also reported during these experiments, which would exaggerate the D/H increase under Martian atmospheric conditions. This trend of dehydration with a strong D/H increase is visible in chassignite amphibole, with amphibole in the highly shocked chassignite NWA 2737 showing much higher D/H ratios and lower water contents than that in Chassigny (figure 3*c* [95]). Shergottite feldspathic glass shows an increase in D/H ratio with increasing shock—ALHA 77005 is the most shocked shergottite [98] and contains glass with the highest D/H ratios, whereas Zagami and Shergotty are two of the least shocked and contain glass with lower D/H ratios (figure 3*d*). However, in contrast to hydrous amphibole, feldspathic glass contains much lower amounts of water, meaning hydrogen implantation overprints any hydrogen loss through devolatilization, and the overall water abundance increases with increasing shock.

The D/H ratio of MI glass follows a different trend from that of feldspathic glass, largely because the former contains more water (figure 3*e*). The D/H ratios are low (δD ≤ 275‰) in the MI glass of Chassigny [90], as well as the depleted and intermediate shergottites Yamato 098459 and ALHA 77005, respectively [58,90]. These low D/H ratios are probably representative of the Martian mantle. By contrast, MI glass in the enriched shergottites GRV 020090 and LAR 06319 contains highly elevated D/H ratios (δD 3386–6034‰ [58,99]). Based on the light rare-earth-element abundance of LAR 06319 melt inclusions, Basu Sarbadhikari *et al*. [100] reported that this meteorite probably derived its enrichment properties from partial melting of an enriched and oxidized mantle reservoir. Thus, the enriched component in these shergottites must have been rich in water equilibrated with the Martian atmosphere. Such enrichment explains why apatite in the depleted shergottite QUE 94201 contains lower D/H ratios than apatite in the enriched shergottites (figure 3*b*).

The D/H ratio of erupting melts on Mars could also be increased by assimilation of crustal material rich in atmospheric hydrogen, such as soil, sediments or ice. Based on sulfur isotope ratios, the shergottite Los Angeles is reported to contain assimilated crustal material, as are Nakhla and ALH 84001 [101]. However, Los Angeles is an enriched shergottite, so its parental melt would have had high D/H ratios prior to any assimilation. Indeed, other enriched shergottites that have not assimilated crustal material (e.g. Shergotty and Zagami [101]), contain apatite with similar D/H ratios to that in Los Angeles (figure 3*b*). Therefore, crustal assimilation does not appear to have had a significant effect on apatite D/H ratio in this meteorite, probably because the melt and assimilant had similar D/H ratios. Nakhla apatite has low D/H ratios reported to represent the Martian mantle [57]. Incorporation of atmosphere-equilibrated assimilant water should have increased the D/H ratio of these apatites, indicating that the Nakhla assimilant did not contain abundant water. ALH 84001 apatite D/H ratios appear to be elevated from the range of the mantle because of degassing. However, only two apatite grains have been measured in this meteorite (δD 751‰ and 2998‰; [90] and [102], respectively), hence much more data are needed before any conclusions can be drawn about the effects of assimilation on the water content of ALH 84001.

### (e) The Earth

A range of D/H ratio values are found on Earth (table 1). The hydrological cycle fractionates hydrogen, creating glacial ice (standard Greenland Ice Sheet Precipitation (GISP) δD = –190‰ [53]; Standard Light Antarctic Precipitation 2 δD = −427.5‰ [103]), ocean water (VSMOW δD = 0‰ [52]) and fresh water (δD = 0 to −300‰ [51]) reservoirs. In contrast to Mars, the Earth's atmospheric and mantle water reservoirs are not kept separate, because subduction provides a means to mix surface water back into the mantle. This mixing produces a variation in mantle δD from –126‰ to +46‰ via slab dehydration and sediment recycling [104,105]. Mid-ocean ridge basalt (MORB) source D/H ratios are more uniformly mixed, forming a narrow range of δD −60 ± 5‰ [54]. However, none of these reservoirs are likely to represent the Earth's original D/H ratio.

The Earth's atmosphere is not a closed system. Experimentally based chemical models suggest Jeans escape could have caused an increase in the Earth's atmospheric D/H ratio of between a factor of 2 and 9 since the formation of the planets [106]. Plate tectonic mixing ensures this change has been incorporated into the mantle. In addition, collisions with hydrogen-bearing planetesimals or cometary material after the Earth's accretion could have altered the D/H ratio of the planet's surface and upper mantle [107]. Therefore, to determine the Earth's original D/H ratio a reservoir that has been completely unaffected by these surface and upper mantle changes is required.

Although alternative theories exist (e.g. [108]), most studies suggest that high ^3^He/^4^He ratios in some ocean island basalts indicate the existence of relatively undegassed regions in the deep mantle compared with the upper mantle, which retain a greater proportion of their primordial He [109,110]. Early Tertiary (60 Myr) picrites from Baffin Island and west Greenland, which represent volcanic rocks from the proto/early Iceland mantle plume, contain the highest recorded terrestrial ^3^He/^4^He ratios [109,110]. These picrites also have Pb and Nd isotopic ratios consistent with primordial mantle ages (4.45–4.55 Ga) [111], indicating the persistence of an ancient, isolated reservoir in the mantle. The undegassed and primitive nature [112] of this reservoir means that it could preserve the Earth's initial D/H ratio. Indeed, the D/H ratios of MI glass in these picrites extend lower than any previously measured mantle values (δD −97‰ to −218‰ [55]).

## 3. Do the measurements fit the models?

Measurements of meteorites from Vesta, C-type asteroids (carbonaceous chondrites) and Mars, along with terrestrial primitive deep mantle material, indicate the initial D/H ratios of water in these planetary bodies lie between approximately δD +200‰ and −590‰ [44,49,55,57,58]. This range could be narrowed if the most negative values from the Martian mantle (δD approx. −100‰ [57,90]) are assumed to be the most representative (with the least input from the D-enriched Martian atmosphere). In addition, the CR chondrites have more positive water D/H ratios than the other C-chondrite groups, which may be a product of oxidation rather than a reflection of the original parent body D/H ratio [113,114]. If this is the case, the range of inner Solar System water D/H can be reduced to approximately δD −100‰ to −590‰. Therefore, compared with the outer Solar System (e.g. [32]), the water D/H ratios of inner Solar System materials appear relatively homogeneous.

In order to determine whether the terrestrial planets sourced their volatiles from C-chondrites, the bulk D/H of these chondrites should be considered, including contributions from organics and possibly nebular hydrogen. The range of C-chondrite bulk D/H is considerably wider than that for just H_2_O, and stretches to much more positive values (δD = −229‰ to +2150‰ [48,49]). For the CI and CM chondrites at least, the water/metal ratios appear to have been high, based on bulk H and C contents [115] and the degree of aqueous alteration (e.g. [116–119]). Hence, hydrogen isotopic fractionation due to oxidation should not have been significant on these parent bodies. C-chondrite parent bodies (C-type asteroids) could originally have had lower bulk δD values if they lost significant water/ice during and after accretion. The heat of accretion could have caused expulsion of water from the warm interiors to the surface/near surface, where it would re-freeze. These ice-rich surfaces may have been stripped off over time by impacts and sublimation (C. Alexander 2017, personal communication). Heating, freezing and sublimation all preferentially leave behind the heavier hydrogen isotope, meaning residual parent body water would become D-enriched (increased δD value). Organics would become similarly enriched via isotopic exchange. There is remote-sensing evidence for ice at the surface of at least some C-type asteroids today [120,121]. However, in the absence of sample return or *in situ* exploration, this theory remains speculative.

Based on observations of extrasolar planetary discs, it is thought that Jupiter and Saturn formed a few million years after the formation of the protoplanetary disc [122]. This places gas giant formation and migration after the period of hydrogen isotope equilibration in the disc [16] and the formation of the chondritic parent bodies [35] (figure 1). Gas-driven inward and outward migration of the giant planets is estimated to have occurred on a time scale of hundreds of thousands of years [123]. The terrestrial planets' characteristics, including Mars' relatively small mass, are best reproduced if these planets formed within a disc that had an outer edge at 1 AU [124,125]. These conditions can be reproduced by Jupiter's inward migration to 1.5 AU and subsequent outward migration—the ‘Grand tack’ model of Solar System formation [126]. Inward migration scatters and mixes S- and C-type asteroids (the parent bodies of O- and C-chondrites, respectively) within this 1 AU terrestrial planet-forming region. S-type asteroids from 1 to 3 AU are reported to make up most of this mixed material [126]. If oxidation occurred before terrestrial planet accretion the water content of the S-type asteroids would be much lower, and the D/H ratio of the remaining water much higher (δD = 798–1209‰ [49]), than that measured for the terrestrial planets. Complete oxidation of the metal in OCs is estimated to take hundreds to 10 000 years [61], with several million years required before this for parent body accretion and heating to the point that oxidation can take place. However, the Grand tack model indicates that the terrestrial planets accreted much later than chondrites, 30–50 Myr after the formation of Ca,Al-rich inclusions [126,127]. In support of this time scale, W isotope data from lunar samples suggest that the Earth–Moon system formed approximately 30–100 Ma after the formation of the Solar System [128]. Therefore, OC oxidation does appear to have occurred prior to terrestrial planet formation, hence OC's D/H ratio would have been high at the time of planetary accretion. This problem can be overcome if a significant proportion of C-chondrite material accreted during terrestrial planet formation. As the OCs are extremely water-poor compared with the C-chondrites, the latter's D/H could overprint that of the OCs—Semarkona is by far the most water-rich OC with 0.09–0.1 wt% H, versus 0.17–1.36 wt% H for the C-chondrites [49].

Based on the Earth's bulk elemental and isotopic composition, numerous studies have attempted to recreate the Earth from different types and proportions of chondritic meteorites (e.g. [129]). However, none of these studies have been able to successfully recreate the abundances of all elements. Nitrogen isotope ratios (^15^N/^14^N) appear to be relatively homogeneous between Vesta, the Moon, Mars and the Earth, but the C-chondrites, particularly the CRs and CMs, have heavier N isotope ratios ([44,130] and references therein). In addition, terrestrial noble gas (Ne and Xe) signatures from primitive mantle sources suggest the presence of a solar component [131,132]. Therefore, accretion of some nebula gas or solar wind is the only way to explain the bulk Earth, Mars (and probably the other terrestrial planet) compositions.

Protosolar nebula water adsorption has been suggested as an alternative theory for the source of inner Solar System planetary body water. The temperature was high at 1 AU during the early Solar System, but 1000–500 K would still allow adsorption of 25–300% of the Earth's ocean water onto fractal grains during the Earth's accretion [133]. Asaduzzaman *et al*. [134] used density functional theory calculations to determine the amount of water that olivine surfaces could retain in the solar nebula. These authors found that water can be retained on adsorption surfaces at temperatures up to 900 K. In addition, if enough water is present in the nebula it may penetrate beyond the olivine grain surface to produce serpentine and brucite. This mechanism would be valid for all inner Solar System rocky materials. However, current models have been unable to accurately reproduce the D/H ratios of these inner Solar System bodies, as the protosolar nebula has a much lower D/H ratio than the range measured in inner Solar System meteorites (δD ≈ −870‰ [38]). In addition, it is unlikely that protosolar adsorption is a valid mechanism for the delivery of other volatile elements (e.g. C, N and noble gases).

## 4. Summary

Once the effects of secondary processes are removed, the original hydrogen isotope ratios of water in the Earth, Mars and Vesta, along with the C- and S-type asteroids, cover a relatively narrow range (approx. δD −100‰ to −590‰). This relative water homogeneity indicates that these materials shared a water source. The differentiated bodies (the Earth, Mars and Vesta) could have sourced their water from the C- and S-type asteroids, as the latter formed prior to terrestrial planet accretion. Oxidation of S-type asteroids almost certainly occurred prior to terrestrial planet accretion in the early Solar System, causing an increase in the bulk rock D/H ratio. However, the low water content of the S-type asteroids means their high D/H ratio could have been overprinted, at least in part, by the low D/H ratio of the C-type asteroids, which have much higher water contents. This simplistic view is complicated by the fact that no combination of chondrite meteorite compositions (as a proxy for parental asteroid compositions) has been able to recreate the bulk Earth. In particular, nitrogen and noble gases suggest that solar wind or nebula gas played a role in the Earth's formation. Inner Solar System nebula hydrogen gas (H_2_) or solar wind would have had a low D/H ratio at the time of terrestrial planet formation, thus this addition could have further overprinted the high D/H ratios of the S-type asteroid component in the terrestrial planets.

## Supplementary Material

SM_DH of Martian igneous minerals
